# *tert*-Butyl *N*-{(1*S*)-1-[(2,4-dihy­droxy­benzyl­idene)hydrazinecarbon­yl]-2-hy­droxy­eth­yl}carbamate ethanol monosolvate

**DOI:** 10.1107/S1600536811003795

**Published:** 2011-02-09

**Authors:** Alessandra C. Pinheiro, Marcus V. N. de Souza, Edward R. T. Tiekink, Solange M. S. V. Wardell, James L. Wardell

**Affiliations:** a Fundação Oswaldo Cruz, Instituto de Tecnologia em Fármacos – Farmanguinhos, R. Sizenando Nabuco, 100, Manguinhos, 21041-250, Rio de Janeiro, RJ, Brazil; b Department of Chemistry, University of Malaya, 50603 Kuala Lumpur, Malaysia; c CHEMSOL, 1 Harcourt Road, Aberdeen AB15 5NY, Scotland; d Centro de Desenvolvimento Tecnológico em Saúde (CDTS), Fundação Oswaldo Cruz (FIOCRUZ), Casa Amarela, Campus de Manguinhos, Av. Brasil 4365, 21040-900 Rio de Janeiro, RJ, Brazil

## Abstract

The mol­ecule of the title ethanol solvate, C_15_H_21_N_3_O_6_·C_2_H_6_O, adopts a curved shape; the conformation about the imine bond [N=N = 1.287 (3) Å] is *E*. The amide residues occupy positions almost orthogonal to each other [dihedral angle = 85.7 (2)°]. In the crystal, a network of O—H⋯O, O—H⋯N and N—H⋯O hydrogen bonds leads to the formation of supra­molecular arrays in the *ab* plane with the ethanol mol­ecules lying to the periphery on either side. Disorder in the solvent ethanol mol­ecule was evident with two positions being resolved for the C atoms [site occupancy of the major component = 0.612 (10)].

## Related literature

For background to the use of l-serine derivatives in anti-tumour therapy, see: Jiao *et al.* (2009 ▶); Yakura *et al.* (2007 ▶); Takahashi *et al.* (1988 ▶); Sin *et al.* (1998 ▶). For background to *N*-acyl­hydrazone derivatives from l-serine for anti-tumour testing, see: Rollas & Küçükgüzel (2007 ▶); Terzioğlu & Gürsoy (2003 ▶). For related structures, see: Pinheiro *et al.* (2010 ▶); de Souza *et al.* (2010 ▶).
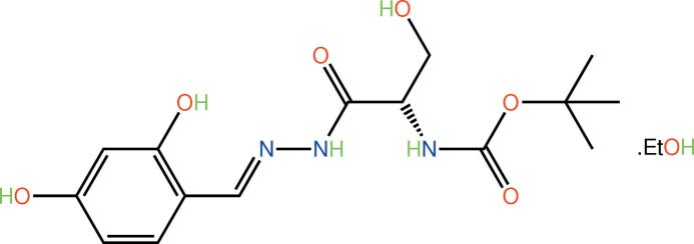


## Experimental

### 

#### Crystal data

C_15_H_21_N_3_O_6_·C_2_H_6_O
*M* *_r_* = 385.42
Monoclinic, 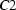

*a* = 17.4054 (4) Å
*b* = 8.7266 (2) Å
*c* = 15.0105 (4) Å
β = 122.219 (2)°
*V* = 1928.87 (8) Å^3^
*Z* = 4
Mo *K*α radiation
μ = 0.10 mm^−1^
*T* = 120 K
0.16 × 0.14 × 0.06 mm

#### Data collection

Bruker–Nonius Roper CCD camera on κ-goniostat diffractometer
Absorption correction: multi-scan (*SADABS*; Sheldrick, 2007 ▶) *T*_min_ = 0.897, *T*_max_ = 1.000
19885 measured reflections
2369 independent reflections
2303 reflections with *I* > 2σ(*I*)
*R*_int_ = 0.041

#### Refinement

*R*[*F* ^2^ > 2σ(*F* ^2^)] = 0.036
*wR*(*F* ^2^) = 0.099
*S* = 1.06
2369 reflections
271 parameters
7 restraints
H atoms treated by a mixture of independent and constrained refinement
Δρ_max_ = 0.61 e Å^−3^
Δρ_min_ = −0.33 e Å^−3^

### 

Data collection: *COLLECT* (Hooft, 1998 ▶); cell refinement: *DENZO* (Otwinowski & Minor, 1997 ▶) and *COLLECT*; data reduction: *DENZO* and *COLLECT*; program(s) used to solve structure: *SHELXS97* (Sheldrick, 2008 ▶); program(s) used to refine structure: *SHELXL97* (Sheldrick, 2008 ▶); molecular graphics: *ORTEP-3* (Farrugia, 1997 ▶) and *DIAMOND* (Brandenburg, 2006 ▶); software used to prepare material for publication: *publCIF* (Westrip, 2010 ▶).

## Supplementary Material

Crystal structure: contains datablocks global, I. DOI: 10.1107/S1600536811003795/hb5793sup1.cif

Structure factors: contains datablocks I. DOI: 10.1107/S1600536811003795/hb5793Isup2.hkl

Additional supplementary materials:  crystallographic information; 3D view; checkCIF report

## Figures and Tables

**Table 1:** Hydrogen-bond geometry (Å, °)

*D*—H⋯*A*	*D*—H	H⋯*A*	*D*⋯*A*	*D*—H⋯*A*
O1—H1*o*⋯N1	0.86 (3)	1.89 (3)	2.643 (3)	147 (3)
N2—H2*n*⋯O3^i^	0.86 (3)	1.91 (2)	2.760 (2)	171 (2)
O2—H2*o*⋯O5^ii^	0.83 (3)	1.86 (3)	2.669 (3)	165 (3)
N3—H3*n*⋯O4^iii^	0.86 (3)	2.08 (3)	2.926 (3)	173 (2)
O4—H4*o*⋯O7^iv^	0.83 (1)	1.94 (2)	2.761 (3)	167 (3)
O7—H7*o*⋯O2^v^	0.84 (1)	2.05 (2)	2.858 (2)	162 (4)

Symmetry codes: (i) 
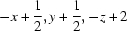
; (ii) 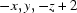; (iii) 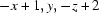; (iv) 
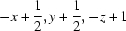
; (v) 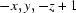.

**Fig. 1.**
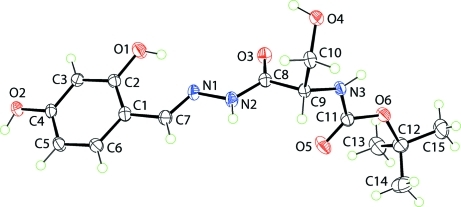
The molecular structure of (I) showing displacement ellipsoids at the 50% probability level. The ethanol molecule of solvation has been omitted.

**Fig. 2.**
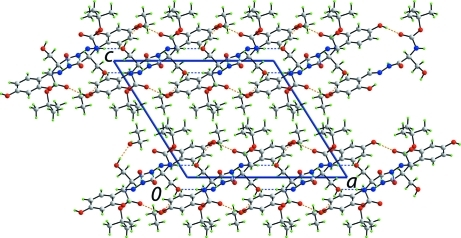
A view in projection down the b axis of the stacking of two-dimensional supramolecular arrays in the ab plane in (I) with the O—H···O and N—H···O hydrogen bonding shown as orange and blue dashed lines, respectively.

## supplementary crystallographic information

### Comment

Several *L*-serine derivatives have been found to have potential in anti-tumour therapy, for example, conagenin, a naturally occurring serine derivative, was shown to improve the anti-tumour efficacy of adriamycin and mitomycin C against murine leukemias (Jiao *et al.*, 2009; Yakura *et al.*, 2007). Other *L*-serine derivatives reported as potential new anti-tumour agents include the antibiotic thrazarine, which sensitizes tumour cells to macrophage-mediated cytolysis (Takahashi *et al.*, 1988), and eponemycin, an immunomodulator, which plays a crucial role in tumour progression and metastases by supplying essential nutrients to B16 melanoma cells (Sin *et al.*, 1998). Following on from such reports, we have synthesized some *N*-acylhydrazones derivatives from *L*-serine to use in anti-tumour testing. The choice of *N*-acylhydrazonyl derivatives was suggested by publications indicating that compounds with such groups can aid anti-tumoural activities (Rollas *et al.*, 2007; Terzioğlu *et al.*, 2003). In continuation of on-going structural studies of these compounds (Pinheiro *et al.*, 2010; de Souza *et al.*, 2010), we now report the structure of the ethanol solvate of *tert*-butyl (1*S*)-2-[2-(2,4-dihydroxybenzylidene)hydrazino]-1-(hydroxymethyl)-2-oxoethylcarbamate, (I).

Although the absolute structure of (I), Fig. 1, could not be determined experimentally, the assignment of the *S*-configuration at the C9 atom is based on a starting reagent. The overall conformation of the molecule is curved with the major kink occurring at the C9 atom. The dihydroxybenzene ring is slightly twisted out of the plane of the hydrazine residue with the C2—C1—C7—N1 torsion angle being -8.2 (3) °. The conformation about the N1—C7 imine bond [1.287 (3) Å] is *E*. Each of the carbonyl groups is diagonally opposite the amine group and the dihedral angle formed between the two amide residues is 85.7 (2) °.

As expected with four hydroxyl and two amine donors, there is significant hydrogen bonding operating in the crystal structure, Table 1. While the O1-hydroxy group forms an intramolecular O–H···N hydrogen bond with the hydrazine-N1 atom, the remaining interactions are intermolecular in nature. The O2-hydroxy group forms an O—H···O hydrogen bond with the O5-carbonyl, and the O3-hydroxyl group linked to the chiral centre is connected to the ethanol molecule which in turn forms a hydrogen bond to the O2-hydroxyl group. The N2-amine is connected to the O3-carbonyl and the N3-amine forms a hydrogen bond with the O4-hydroxyl. The result of the hydrogen bonding is the formation of layers of molecules in the *ab* plane sandwiched by ethanol molecules. The layers stack along the *c* axis, Fig. 2.

### Experimental

To a stirred solution of *tert*-butyl (1*S*)-2-hydrazino-1-(hydroxymethyl)-2-oxoethylcarbamate (1.0 mmol) in ethanol (10 ml) at room temperature was added 2,4-dihydroxybenzaldehyde (1.05 mmol). The reaction mixture was stirred for 4 h. at 1073 K and concentrated under reduced pressure. The residue was purified by washing with cold ethanol (3 *x* 10 ml), affording the target molecule in 74% yield, m.pt. 423–424 K. The colourless block used in the structure determination was grown from EtOH. ^1^H NMR (500 MHz, DMSO-d6) δ (p.p.m.): 11.50 (1*H*, s, NHN), 11.30 (1*H*, s), 9.92 (1*H*, s), 8.30 (1*H*, s, N=CH), 7.26 (1*H*, d, J = 8.4 Hz, H6), 6.80 (1*H*, d, J = 7.7 Hz, NHCH), 6.35–6.30 (1*H*, m, H5), 6.29 (1*H*, s, H3), 4.95 (1*H*, s, OH), 4.02 (1*H*, m, CH), 3.70–3.50 (2*H*, m, CH_2_OH); 1.39 (9*H*, s, (CH_3_)_3_C). ^13^C NMR (125 MHz, DMSO-d6) δ (p.p.m.): 170.8, 160.3, 157.9, 155.2, 141.8, 128.0, 110.4, 107.6, 102.3, 78.0, 61.1, 53.9, 28.2. IR (cm^-1^; KBr): 3200 (O—H), 1678 (COCH and COO). EM/ESI: [M—H]: 338.3.

### Refinement

The C-bound H atoms were geometrically placed (C–H = 0.95–0.99 Å) and refined as riding with *U*_iso_(H) = 1.2–1.5*U*_eq_(C). The O– and N-bound H atoms were located from a difference map and refined with the distance restraints O–H = 0.84 ± 0.01 and N–H = 0.86±0.01 Å, and with *U*_iso_(H) = *zU*_eq_(carrier atom); *z* = 1.5 for O and *z* = 1.2 for N. Disorder was resolved in the solvent ethanol molecule in that two distinct positions were discerned for the C atoms. From fractional anisotropic refinement, the major component had a site occupancy factor = 0.612 (10). In the absence of significant anomalous scattering effects, 2067 Friedel pairs were averaged in the final refinement. However, the absolute configuration was assigned on the basis of the chirality of the *L*-serine starting material. The maximum and minimum residual electron density peaks of 0.61 and 0.33 e Å^-3^, respectively, were located 0.42 Å and 0.37 Å from the H6 and H16*a* atoms, respectively.

### Crystal data

**Table:**

C_15_H_21_N_3_O_6_·C_2_H_6_O	*F*(000) = 824
*M**_r_* = 385.42	*D*_x_ = 1.327 Mg m^−^^3^
Monoclinic, *C*2	Mo *K*α radiation, λ = 0.71073 Å
Hall symbol: C 2y	Cell parameters from 4327 reflections
*a* = 17.4054 (4) Å	θ = 2.9–27.5°
*b* = 8.7266 (2) Å	µ = 0.10 mm^−^^1^
*c* = 15.0105 (4) Å	*T* = 120 K
β = 122.219 (2)°	Block, colourless
*V* = 1928.87 (8) Å^3^	0.16 × 0.14 × 0.06 mm
*Z* = 4	

### Data collection

**Table:**

Bruker–Nonius Roper CCD camera on κ-goniostat diffractometer	2369 independent reflections
Radiation source: Bruker-Nonius FR591 rotating anode CCD	2303 reflections with *I* > 2σ(*I*)
graphite	*R*_int_ = 0.041
Detector resolution: 9.091 pixels mm^-1^	θ_max_ = 27.5°, θ_min_ = 3.2°
φ and ω scans	*h* = −22→22
Absorption correction: multi-scan (*SADABS*; Sheldrick, 2007)	*k* = −11→11
*T*_min_ = 0.897, *T*_max_ = 1.000	*l* = −19→19
19885 measured reflections	

### Refinement

**Table:**

Refinement on *F*^2^	Primary atom site location: structure-invariant direct methods
Least-squares matrix: full	Secondary atom site location: difference Fourier map
*R*[*F*^2^ > 2σ(*F*^2^)] = 0.036	Hydrogen site location: inferred from neighbouring sites
*wR*(*F*^2^) = 0.099	H atoms treated by a mixture of independent and constrained refinement
*S* = 1.06	*w* = 1/[σ^2^(*F*_o_^2^) + (0.0606*P*)^2^ + 1.0679*P*] where *P* = (*F*_o_^2^ + 2*F*_c_^2^)/3
2369 reflections	(Δ/σ)_max_ = 0.001
271 parameters	Δρ_max_ = 0.61 e Å^−^^3^
7 restraints	Δρ_min_ = −0.33 e Å^−^^3^

### Special details

**Table:**

Geometry. All s.u.'s (except the s.u. in the dihedral angle between two l.s. planes) are estimated using the full covariance matrix. The cell s.u.'s are taken into account individually in the estimation of s.u.'s in distances, angles and torsion angles; correlations between s.u.'s in cell parameters are only used when they are defined by crystal symmetry. An approximate (isotropic) treatment of cell s.u.'s is used for estimating s.u.'s involving l.s. planes.
Refinement. Refinement of *F*^2^ against ALL reflections. The weighted *R*-factor *wR* and goodness of fit *S* are based on *F*^2^, conventional *R*-factors *R* are based on *F*, with *F* set to zero for negative *F*^2^. The threshold expression of *F*^2^ > 2σ(*F*^2^) is used only for calculating *R*-factors(gt) *etc*. and is not relevant to the choice of reflections for refinement. *R*-factors based on *F*^2^ are statistically about twice as large as those based on *F*, and *R*- factors based on ALL data will be even larger.

### Fractional atomic coordinates and isotropic or equivalent isotropic displacement parameters (Å^2^)

**Table:**

	*x*	*y*	*z*	*U*_iso_*/*U*_eq_	Occ. (<1)
O1	−0.01014 (11)	0.6314 (2)	0.76103 (14)	0.0306 (4)	
H1o	0.0421 (13)	0.674 (4)	0.796 (2)	0.046*	
O2	−0.30367 (9)	0.6766 (2)	0.69839 (12)	0.0259 (3)	
H2o	−0.3330 (19)	0.728 (3)	0.717 (2)	0.039*	
O3	0.27544 (10)	0.67257 (19)	0.95473 (12)	0.0251 (3)	
O4	0.39535 (10)	0.8716 (2)	0.89717 (11)	0.0266 (3)	
H4o	0.3474 (13)	0.853 (4)	0.8402 (14)	0.040*	
O5	0.39849 (11)	0.7949 (2)	1.22519 (13)	0.0333 (4)	
O6	0.53955 (10)	0.7219 (2)	1.26444 (11)	0.0267 (4)	
N1	0.11580 (11)	0.8217 (2)	0.89800 (13)	0.0219 (4)	
N2	0.20132 (11)	0.8878 (2)	0.95418 (14)	0.0221 (4)	
H2n	0.2041 (18)	0.9793 (16)	0.976 (2)	0.027*	
N3	0.44121 (11)	0.8160 (2)	1.10779 (13)	0.0206 (4)	
H3n	0.4884 (12)	0.824 (3)	1.104 (2)	0.025*	
C1	−0.04087 (13)	0.8468 (3)	0.83868 (15)	0.0209 (4)	
C2	−0.06834 (13)	0.7128 (2)	0.77631 (15)	0.0204 (4)	
C3	−0.15725 (13)	0.6607 (3)	0.72779 (15)	0.0214 (4)	
H3	−0.1759	0.5722	0.6843	0.026*	
C4	−0.21852 (13)	0.7390 (3)	0.74340 (15)	0.0205 (4)	
C5	−0.19388 (13)	0.8728 (3)	0.80333 (16)	0.0237 (4)	
H5	−0.2368	0.9262	0.8124	0.028*	
C6	−0.10573 (14)	0.9263 (3)	0.84935 (16)	0.0230 (4)	
H6	−0.0888	1.0186	0.8889	0.028*	
C7	0.05209 (14)	0.9025 (3)	0.89384 (16)	0.0218 (4)	
H7	0.0660	0.9998	0.9273	0.026*	
C8	0.27609 (13)	0.8076 (2)	0.97909 (15)	0.0193 (4)	
C9	0.36197 (12)	0.9053 (2)	1.03518 (15)	0.0192 (4)	
H9	0.3536	0.9876	1.0756	0.023*	
C10	0.37737 (13)	0.9809 (3)	0.95365 (16)	0.0232 (4)	
H10A	0.3228	1.0408	0.9035	0.028*	
H10B	0.4292	1.0528	0.9900	0.028*	
C11	0.45577 (14)	0.7797 (3)	1.20233 (17)	0.0227 (4)	
C12	0.57832 (15)	0.6887 (3)	1.37713 (16)	0.0303 (5)	
C13	0.52980 (18)	0.5534 (4)	1.3883 (2)	0.0379 (6)	
H13A	0.4669	0.5818	1.3629	0.057*	
H13B	0.5608	0.5234	1.4626	0.057*	
H13C	0.5302	0.4672	1.3467	0.057*	
C14	0.5758 (2)	0.8318 (4)	1.4328 (2)	0.0445 (7)	
H14A	0.5988	0.9190	1.4126	0.067*	
H14B	0.6139	0.8166	1.5092	0.067*	
H14C	0.5132	0.8521	1.4127	0.067*	
C15	0.67546 (16)	0.6455 (4)	1.41358 (19)	0.0429 (7)	
H15A	0.6751	0.5548	1.3748	0.064*	
H15B	0.7093	0.6227	1.4891	0.064*	
H15C	0.7045	0.7309	1.4003	0.064*	
O7	0.27404 (12)	0.3530 (2)	0.28565 (16)	0.0403 (4)	
H7o	0.292 (3)	0.443 (2)	0.304 (3)	0.060*	
C16	0.3454 (10)	0.245 (2)	0.3050 (12)	0.0525 (8)	0.612 (10)
H16A	0.3995	0.3029	0.3182	0.063*	0.612 (10)
H16B	0.3241	0.1816	0.2413	0.063*	0.612 (10)
C17	0.3713 (5)	0.1452 (9)	0.3940 (6)	0.0495 (14)	0.612 (10)
H17A	0.3926	0.2073	0.4574	0.074*	0.612 (10)
H17B	0.4200	0.0765	0.4046	0.074*	0.612 (10)
H17C	0.3186	0.0845	0.3801	0.074*	0.612 (10)
C18	0.3420 (13)	0.249 (3)	0.3037 (18)	0.0525 (8)	0.388 (10)
H18A	0.3795	0.2940	0.2789	0.063*	0.388 (10)
H18B	0.3133	0.1547	0.2621	0.063*	0.388 (10)
C19	0.4010 (8)	0.2074 (14)	0.4154 (9)	0.0495 (14)	0.388 (10)
H19A	0.4427	0.2922	0.4538	0.074*	0.388 (10)
H19B	0.4360	0.1155	0.4219	0.074*	0.388 (10)
H19C	0.3637	0.1869	0.4450	0.074*	0.388 (10)

### Atomic displacement parameters (Å^2^)

**Table:**

	*U*^11^	*U*^22^	*U*^33^	*U*^12^	*U*^13^	*U*^23^
O1	0.0239 (7)	0.0322 (9)	0.0381 (9)	−0.0014 (7)	0.0182 (7)	−0.0059 (7)
O2	0.0155 (7)	0.0351 (9)	0.0283 (8)	−0.0018 (6)	0.0124 (6)	−0.0029 (7)
O3	0.0231 (7)	0.0197 (7)	0.0301 (7)	−0.0008 (6)	0.0126 (6)	−0.0001 (6)
O4	0.0205 (7)	0.0384 (9)	0.0223 (7)	0.0012 (7)	0.0123 (6)	0.0005 (7)
O5	0.0274 (8)	0.0464 (10)	0.0352 (8)	0.0105 (8)	0.0227 (7)	0.0136 (8)
O6	0.0211 (7)	0.0410 (10)	0.0188 (7)	0.0088 (7)	0.0111 (6)	0.0070 (7)
N1	0.0152 (7)	0.0239 (9)	0.0251 (8)	−0.0026 (7)	0.0097 (7)	−0.0006 (7)
N2	0.0169 (8)	0.0203 (9)	0.0271 (8)	−0.0025 (7)	0.0104 (7)	−0.0017 (7)
N3	0.0154 (7)	0.0261 (9)	0.0205 (8)	0.0034 (7)	0.0097 (6)	0.0031 (7)
C1	0.0180 (8)	0.0233 (10)	0.0208 (8)	0.0006 (8)	0.0100 (7)	0.0034 (8)
C2	0.0187 (8)	0.0238 (11)	0.0196 (8)	0.0019 (8)	0.0109 (7)	0.0035 (8)
C3	0.0193 (9)	0.0250 (10)	0.0192 (8)	−0.0005 (8)	0.0098 (7)	−0.0001 (8)
C4	0.0147 (8)	0.0272 (10)	0.0188 (8)	0.0007 (8)	0.0084 (7)	0.0042 (8)
C5	0.0188 (9)	0.0300 (12)	0.0246 (9)	0.0041 (8)	0.0132 (8)	0.0026 (8)
C6	0.0218 (9)	0.0245 (10)	0.0247 (9)	0.0009 (8)	0.0137 (8)	−0.0004 (8)
C7	0.0199 (9)	0.0218 (10)	0.0231 (9)	−0.0018 (8)	0.0109 (8)	0.0000 (8)
C8	0.0184 (9)	0.0201 (10)	0.0200 (8)	0.0001 (8)	0.0106 (7)	0.0035 (7)
C9	0.0158 (8)	0.0190 (9)	0.0216 (8)	0.0009 (7)	0.0091 (7)	0.0003 (7)
C10	0.0175 (9)	0.0248 (10)	0.0265 (10)	−0.0001 (8)	0.0113 (8)	0.0034 (8)
C11	0.0200 (9)	0.0255 (10)	0.0230 (9)	0.0020 (8)	0.0116 (8)	0.0028 (8)
C12	0.0291 (11)	0.0417 (13)	0.0195 (9)	0.0073 (10)	0.0127 (8)	0.0072 (9)
C13	0.0358 (12)	0.0448 (15)	0.0369 (13)	0.0103 (11)	0.0219 (11)	0.0141 (11)
C14	0.0570 (17)	0.0470 (16)	0.0246 (11)	0.0099 (14)	0.0184 (12)	0.0012 (11)
C15	0.0255 (11)	0.070 (2)	0.0263 (11)	0.0118 (12)	0.0096 (9)	0.0146 (12)
O7	0.0275 (8)	0.0350 (10)	0.0458 (10)	0.0023 (8)	0.0110 (8)	0.0098 (9)
C16	0.0547 (19)	0.0421 (17)	0.0537 (18)	0.0098 (15)	0.0241 (16)	0.0009 (14)
C17	0.047 (3)	0.045 (4)	0.054 (3)	0.014 (2)	0.025 (3)	0.012 (3)
C18	0.0547 (19)	0.0421 (17)	0.0537 (18)	0.0098 (15)	0.0241 (16)	0.0009 (14)
C19	0.047 (3)	0.045 (4)	0.054 (3)	0.014 (2)	0.025 (3)	0.012 (3)

### Geometric parameters (Å, °)

**Table:**

O1—C2	1.353 (3)	C9—H9	1.0000
O1—H1o	0.86 (3)	C10—H10A	0.9900
O2—C4	1.372 (2)	C10—H10B	0.9900
O2—H2o	0.83 (3)	C12—C13	1.512 (4)
O3—C8	1.232 (3)	C12—C14	1.516 (4)
O4—C10	1.417 (3)	C12—C15	1.521 (3)
O4—H4o	0.833 (10)	C13—H13A	0.9800
O5—C11	1.222 (3)	C13—H13B	0.9800
O6—C11	1.342 (2)	C13—H13C	0.9800
O6—C12	1.478 (2)	C14—H14A	0.9800
N1—C7	1.287 (3)	C14—H14B	0.9800
N1—N2	1.386 (2)	C14—H14C	0.9800
N2—C8	1.343 (3)	C15—H15A	0.9800
N2—H2n	0.855 (10)	C15—H15B	0.9800
N3—C11	1.340 (3)	C15—H15C	0.9800
N3—C9	1.447 (2)	O7—C18	1.400 (9)
N3—H3n	0.86 (3)	O7—C16	1.460 (8)
C1—C6	1.405 (3)	O7—H7O	0.842 (10)
C1—C2	1.412 (3)	C16—C17	1.450 (12)
C1—C7	1.453 (3)	C16—H16A	0.9900
C2—C3	1.389 (3)	C16—H16B	0.9900
C3—C4	1.388 (3)	C17—H17A	0.9800
C3—H3	0.9500	C17—H17B	0.9800
C4—C5	1.394 (3)	C17—H17C	0.9800
C5—C6	1.385 (3)	C18—C19	1.47 (2)
C5—H5	0.9500	C18—H18A	0.9900
C6—H6	0.9500	C18—H18B	0.9900
C7—H7	0.9500	C19—H19A	0.9800
C8—C9	1.525 (3)	C19—H19B	0.9800
C9—C10	1.535 (3)	C19—H19C	0.9800
			
C2—O1—H1o	109 (3)	O5—C11—N3	123.4 (2)
C4—O2—H2o	108 (2)	O6—C11—N3	110.28 (17)
C10—O4—H4o	109 (2)	O6—C12—C13	109.8 (2)
C11—O6—C12	122.20 (16)	O6—C12—C14	109.9 (2)
C7—N1—N2	114.81 (18)	C13—C12—C14	113.6 (2)
C8—N2—N1	121.30 (18)	O6—C12—C15	101.66 (17)
C8—N2—H2n	122.1 (18)	C13—C12—C15	110.2 (2)
N1—N2—H2n	116.4 (18)	C14—C12—C15	111.0 (2)
C11—N3—C9	119.41 (16)	C12—C13—H13A	109.5
C11—N3—H3n	116.2 (18)	C12—C13—H13B	109.5
C9—N3—H3n	118.0 (19)	H13A—C13—H13B	109.5
C6—C1—C2	118.39 (18)	C12—C13—H13C	109.5
C6—C1—C7	119.2 (2)	H13A—C13—H13C	109.5
C2—C1—C7	122.35 (18)	H13B—C13—H13C	109.5
O1—C2—C3	117.67 (19)	C12—C14—H14A	109.5
O1—C2—C1	121.84 (18)	C12—C14—H14B	109.5
C3—C2—C1	120.49 (18)	H14A—C14—H14B	109.5
C2—C3—C4	119.5 (2)	C12—C14—H14C	109.5
C2—C3—H3	120.3	H14A—C14—H14C	109.5
C4—C3—H3	120.3	H14B—C14—H14C	109.5
O2—C4—C3	116.6 (2)	C12—C15—H15A	109.5
O2—C4—C5	121.99 (18)	C12—C15—H15B	109.5
C3—C4—C5	121.43 (18)	H15A—C15—H15B	109.5
C6—C5—C4	118.80 (19)	C12—C15—H15C	109.5
C6—C5—H5	120.6	H15A—C15—H15C	109.5
C4—C5—H5	120.6	H15B—C15—H15C	109.5
C5—C6—C1	121.4 (2)	C18—O7—C16	0(3)
C5—C6—H6	119.3	C18—O7—H7o	114 (3)
C1—C6—H6	119.3	C16—O7—H7o	114 (3)
N1—C7—C1	120.9 (2)	O7—C16—C17	112.6 (7)
N1—C7—H7	119.6	O7—C16—H16A	109.1
C1—C7—H7	119.6	C17—C16—H16A	109.1
O3—C8—N2	124.15 (19)	O7—C16—H16B	109.1
O3—C8—C9	123.30 (18)	C17—C16—H16B	109.1
N2—C8—C9	112.44 (18)	H16A—C16—H16B	107.8
N3—C9—C8	112.03 (17)	O7—C18—C19	112.6 (15)
N3—C9—C10	109.30 (16)	O7—C18—H18A	109.1
C8—C9—C10	109.66 (16)	C19—C18—H18A	109.1
N3—C9—H9	108.6	O7—C18—H18B	109.1
C8—C9—H9	108.6	C19—C18—H18B	109.1
C10—C9—H9	108.6	H18A—C18—H18B	107.8
O4—C10—C9	112.07 (18)	C18—C19—H19A	109.5
O4—C10—H10A	109.2	C18—C19—H19B	109.5
C9—C10—H10A	109.2	H19A—C19—H19B	109.5
O4—C10—H10B	109.2	C18—C19—H19C	109.5
C9—C10—H10B	109.2	H19A—C19—H19C	109.5
H10A—C10—H10B	107.9	H19B—C19—H19C	109.5
O5—C11—O6	126.34 (19)		
			
C7—N1—N2—C8	169.49 (18)	N1—N2—C8—C9	176.62 (17)
C6—C1—C2—O1	179.01 (19)	C11—N3—C9—C8	−78.9 (2)
C7—C1—C2—O1	−2.6 (3)	C11—N3—C9—C10	159.32 (19)
C6—C1—C2—C3	−0.6 (3)	O3—C8—C9—N3	−33.9 (3)
C7—C1—C2—C3	177.78 (19)	N2—C8—C9—N3	149.72 (17)
O1—C2—C3—C4	178.56 (18)	O3—C8—C9—C10	87.7 (2)
C1—C2—C3—C4	−1.8 (3)	N2—C8—C9—C10	−88.7 (2)
C2—C3—C4—O2	−176.30 (18)	N3—C9—C10—O4	58.1 (2)
C2—C3—C4—C5	2.7 (3)	C8—C9—C10—O4	−65.0 (2)
O2—C4—C5—C6	177.85 (19)	C12—O6—C11—O5	−9.2 (4)
C3—C4—C5—C6	−1.1 (3)	C12—O6—C11—N3	172.7 (2)
C4—C5—C6—C1	−1.5 (3)	C9—N3—C11—O5	12.9 (4)
C2—C1—C6—C5	2.3 (3)	C9—N3—C11—O6	−168.91 (18)
C7—C1—C6—C5	−176.19 (19)	C11—O6—C12—C13	69.9 (3)
N2—N1—C7—C1	−179.27 (17)	C11—O6—C12—C14	−55.8 (3)
C6—C1—C7—N1	170.21 (19)	C11—O6—C12—C15	−173.4 (2)
C2—C1—C7—N1	−8.2 (3)	C18—O7—C16—C17	−98 (83)
N1—N2—C8—O3	0.3 (3)	C16—O7—C18—C19	52 (82)

### Hydrogen-bond geometry (Å, °)

**Table:**

*D*—H···*A*	*D*—H	H···*A*	*D*···*A*	*D*—H···*A*
O1—H1o···N1	0.86 (3)	1.89 (3)	2.643 (3)	147 (3)
N2—H2n···O3^i^	0.86 (3)	1.91 (2)	2.760 (2)	171 (2)
O2—H2o···O5^ii^	0.83 (3)	1.86 (3)	2.669 (3)	165 (3)
N3—H3n···O4^iii^	0.86 (3)	2.08 (3)	2.926 (3)	173 (2)
O4—H4o···O7^iv^	0.83 (1)	1.94 (2)	2.761 (3)	167 (3)
O7—H7o···O2^v^	0.84 (1)	2.05 (2)	2.858 (2)	162 (4)

Symmetry codes:  (i) −*x*+1/2, *y*+1/2, −*z*+2; (ii) −*x*, *y*, −*z*+2; (iii) −*x*+1, *y*, −*z*+2; (iv) −*x*+1/2, *y*+1/2, −*z*+1; (v) −*x*, *y*, −*z*+1.
